# Acceptability and impact on anthropometry of a locally developed Ready-to-use therapeutic food in pre-school children in Vietnam

**DOI:** 10.1186/1475-2891-12-120

**Published:** 2013-08-15

**Authors:** Tran T Nga, Marie Nguyen, Roger Mathisen, Do TB Hoa, Nguyen H Minh, Jacques Berger, Frank T Wieringa

**Affiliations:** 1 National Institute of Nutrition (NIN), Hanoi, Vietnam; 2 UMR 204 NUTRIPASS « Prevention of Malnutrition and associated pathologies », IRD-UMR2-UMR1, Institute of Research for Development (IRD), Montpellier, France; 3 UNICEF Vietnam, 81A Tran Quoc Toan, Hanoi, Vietnam; 4 UMR204 'Nutripass' IRD/UM2/UM1, Institut de Recherche pour le Développement (IRD), BP 64501-911, avenue d'Agropolis, 34394 MONTPELLIER CEDEX 5, France

**Keywords:** RUTF, Severe acute malnutrition, Anthropometry, Acceptability, Preschool children

## Abstract

**Background:**

In South East Asia, concerns exist about the acceptability of peanut-based Ready-to-Use-Therapeutic-Foods (RUTF) for the treatment of severe acute malnutrition (SAM). Therefore, an alternative, culturally acceptable RUTF made from locally available ingredients and complying with local food traditions and preferences was developed. The current study evaluated its acceptability and impact on anthropometry.

**Methods:**

The study was a randomized, two-arm, cross-over intervention trial to test the acceptability of the local product (bar) against a commercially available, peanut-based RUTF paste (Plumpy’nut^®^). Children (n = 67) from two kindergartens in a rural area of North Vietnam were recruited. The age of the children was between 3 and 5 years.

**Results:**

The Vietnamese RUTF was well-accepted, although overall acceptability was less than of Plumpy’nut^®^, with the latter scoring higher on palatability (P < 0.05). In contrast, reluctance to eat Plumpy’nut^®^ was higher than for the Vietnamese RUTF (P < 0.05). Impact on anthropmetrical indices was similar for both RUTF. The nutritional status of the children who consumed the two RUTF over a 4 week period improved significantly, with a mean weight gain of 0.64 (SD 0.27) Kg, and increases in WHZ and HAZ z-scores of 0.48 (SD 0.30) and 0.05 (SD 0.13) respectively (P < 0.01 both). Weight gain was similar between the 2 products (0.32 kg per 2 weeks for both).

**Conclusions:**

Both the commercial Plumpy’nut^®^ and the local produced RUTF were accepted although the harder consistency of the local product might have caused the lower overall acceptance. The promising increase in nutritional status needs to be confirmed in a controlled trial in children with SAM.

## Introduction

Severe acute malnutrition (SAM) continues to be a major public health problem throughout the developing world, particularly in sub-Saharan Africa and South Asia. An estimated 20 million children million suffer from SAM [1]. SAM is being defined by the World Health Organization as a weight-for-height z-score (WHZ) of < −3.0 or a mid upper arm circumference (MUAC) of <115 mm. Diets deficient both in macronutrients and micronutrients in combination with a high burden of infection are the main underlying causes of child malnutrition. Children with SAM need to be treated with specialized therapeutic diets in combination with diagnosis and management of infections and other complications. In Africa, the development of so-called Ready-to-Use-Therapeutic-Foods (RUTF) has made home-based treatment of SAM in children feasible and is as least as successful as hospital-based treatment [2].

RUTF is a generic term including different types of foods, such as spreads or compressed products, specifically designed for the treatment of SAM. RUTFs have a very high energy density of about 23 kJ/g (5.5 kcal/g) [3], and in general consist of a mixture of milk powder, vegetable oil, sugar, peanut butter and a vitamin-mineral premix. Since RUTF are not water based, bacterial growth is limited in those foods. Therefore they can be used safely at home without refrigeration and even in areas where hygiene conditions are not optimal [1]. The ready-to-use nature of the foods means that considerable time, work, effort and money are spared in the care for SAM cases. International organizations such as WHO, UNICEF and WFP have precisely specified the composition of RUTF.

Many studies, most in Africa, have demonstrated the acceptability and effectiveness of RUTF in the treatment of SAM [4-7]. Thus, the World Health Organization and UNICEF recommend RUTF for the treatment of SAM, both in non-emergency situations and in disaster relief programs [8].

In Asia, the rate of SAM and moderate acute malnutrition (MAM, WHZ < −2) among children under 5 years of age is 17% [9]. Although peanut-based RUTF is also used in Asia, there are only few publications about their acceptability and/or effectiveness. A recent acceptability trial in Cambodia with Plumpy’nut^®^, the most widely used RUTF in Africa, showed that it was not well accepted by Cambodian children, although many other problems with the introduction of Plumpy’nut^®^ in Cambodia, including lack of information and comprehension for both health workers and caregivers, were also identified [10]. However, this failure to successfully implement a RUTF for the treatment of SAM in Cambodia has resulted in concerns in SE-Asia about the acceptability of currently used RUTF’s.

Vietnam is developing guidelines for the 2011–2015 National Action Plan for Infant and Young Child Feeding, including national guidelines for the Integrated Management of Acute Malnutrition (IMAM) for children with SAM. About 7.1% of the children under 5 years of age are wasted in Vietnam, i.e. with a W/H < −2 Z-score [11]. To be able to implement IMAM, community-based treatment of acute malnutrition with RUTFs is essential. To facilitate the implementation of IMAM in communities in Vietnam, the National Institute of Nutrition (NIN) in collaboration with the Institute of Research for Development (IRD) and UNICEF, jointly started developing a RUTF which could be produced locally, and which was adapted to local taste preferences. This paper describes the development and testing of the acceptability of the local developed RUTF in comparison with the conventional therapeutic product Plumpy’nut^®^. In addition, changes in anthropometrical indices during the study are reported.

### Experimental methods

#### Development of RUTF

The composition of the RUTF was based on nutritional qualities of the ingredients, local availability and price. After an initial exploratory phase, the research team decided to develop a RUTF in form of a compressed bar instead of a paste-like product), as this more resembled a popular Vietnamese snack (Banh Dau Xanh or green bean cake), improving the chances for producing an acceptable product. Furthermore, the final composition had to fulfill WHO and UNICEF requirements for RUTF, including an energy density from 5.2 to 5.5 kcal/g, protein from 10 to 12% of total energy (with at least 50% derived from milk products), lipids from 45 to 60% of total energy and a maximum water content of 2.5% [1]. Finally, the composition needed to result in a texture suitable for compressing the product into a soft bar agreeable for consumers. Thus the composition of the final product, which was optimized for above restrictions, contained mung and soy beans, rice, sesame, sugar, whole milk powder, whey protein, vegetable fat, vegetable oil and a premix (Table 1).

**Table 1:** Composition of the Vietnamese RUTF

**Nutrition facts per 100 g**	
Energy	543.48 Kcal
Protein	15.33 g
Lipid	34.67 g
n-6 fatty acids	4.82 g
n-3 fatty acids	1.61 g
Carbohydrate	42.50 g
Moisture content	2.5%
**Ingredients of the premix**	**Quantity/100 g**
**Vitamins**	
Vitamin A	1 mg
Vitamin D	15 μg
Vitamin E	20 mg
Vitamin K	21μg
Vitamin C	50 mg
Vitamin B1	0.3 mg
Vitamin B2	1.4 mg
Vitamin B6	0.5 mg
Vitamin B12	1.5 μg
Folic Acid	0.2 mg
Niacinamide	5 mg
Panthothenic acid	1.49 mg
Biotin	8.3 μg
**Minerals**	
Potassium	700 mg
Magnesium	50 mg
Iron	8 mg
Copper	1 mg
Iodine	19 μg
Zinc	12 mg
Selenium	40 μg

**Figure 1:**
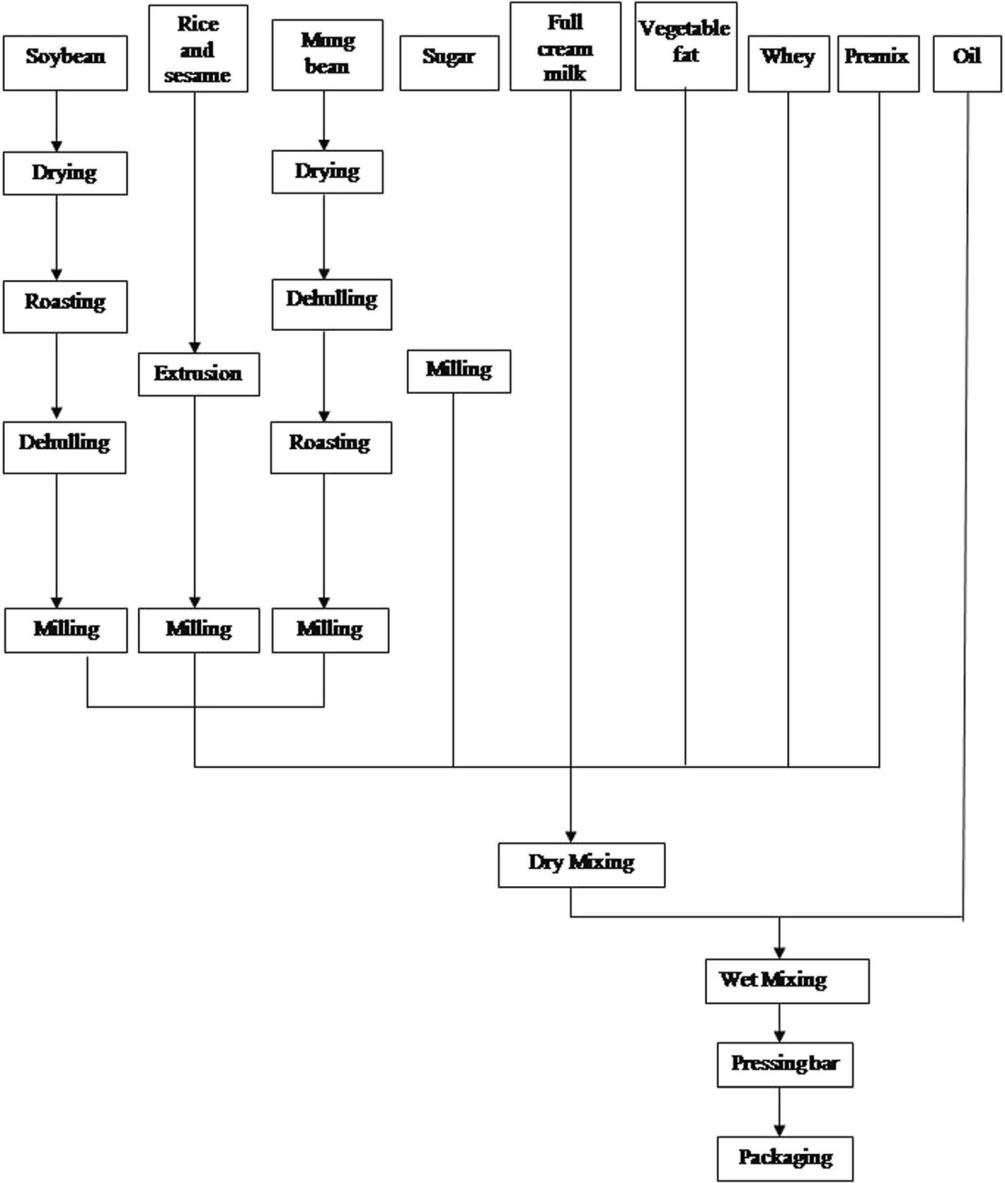
Local RUTF production.

The RUTF was produced at the factory of the Food Technology Department of the NIN, based at the University of Nong Nghiep, Hanoi. This facility is standardized for the production of food items for human consumption. Soya beans and green beans were dried (2 hrs at 110°C) and roasted (1 hr at 115°C) to reduce the water and lysine content and to enhance taste, before being mixed with extruded rice and the other components (Figure 1). The resulting stiff dough was then pressed into a 50 g rectangular bar (8×4×1.5 cm) following the traditional shape of the Vietnamese sweet mung bean flour cakes, at the Cong Ty Co Phan 22 factory in Hanoi. Then 2 bars were packed to make a 100 g sachet. The Vietnamese RUTF was given the name of HEBI (High Energy Bar for IMAM).

#### Acceptability trial

**Figure 2:**
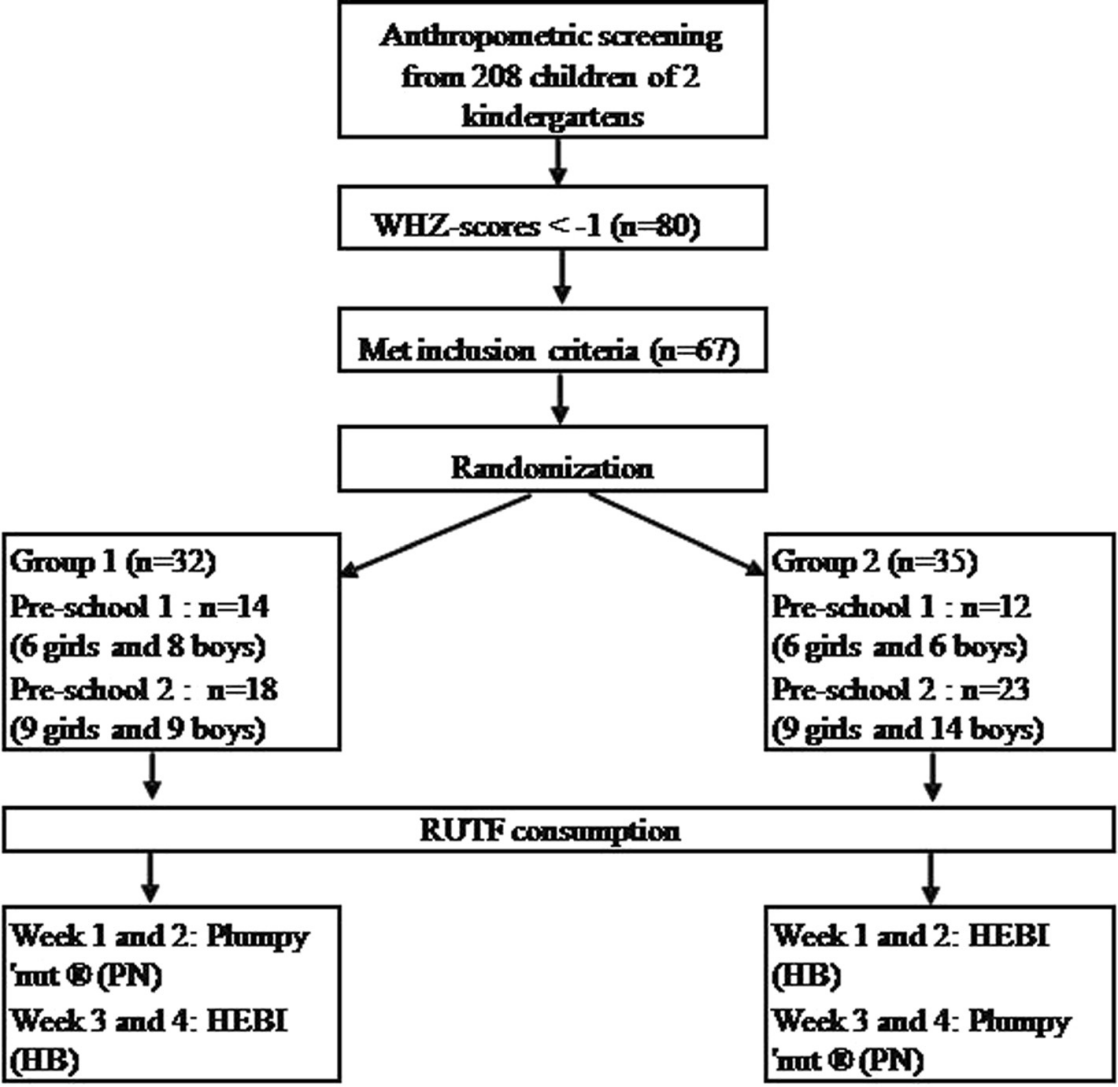
**Study profile: initial screening and enrollment of the children in the study, followed by a 4 weeks trial.**

To assess the acceptability and organoleptic qualities of the product, a trial was conducted to compare HEBI with the commercially available Plumpy’nut^®^ (Nutriset, France). The trial was a randomized, cross-over designed study in which children received both products subsequently for 2 weeks, two times per school day. Before the trial, an initial screening of 208 children in 2 kindergartens was done in June 2010 to enroll children in the study. The kindergartens were located in two different communes, Tien Noi and Trac Van, in Duy Tien district in Ha Nam province, 40 km to the south of Hanoi. The trial was then conducted in July 2010. All children (n = 67) fulfilling the inclusion criteria and for whom written informed consent was obtained were recruited. Inclusion criteria included a WHZ between −3 and −1 z-score and an age between 3 and 6 years.The reason for these selection criteria was that the main outcome of the study was the acceptability of the product, and not impact on anthropometric indices nor the impact on SAM. Assessing acceptability in children <3 yrs of age is difficult, as reliable answers on organoleptic qualities are hard to obtain. The randomization list was made before the start of the study by one researcher not involved in the field work. The subjects were randomly divided in 2 groups of 32 and 35 children, with each group receiving one product during the first two weeks and the other product the second two weeks and vice-versa in the other group. As Plumpy’nut^®^ is a paste and the local RUTF a bar, the study could not be blinded. Figure 2 shows the trial flow chart. As the trial’s main objective was the assessment of the acceptability of the new product and not the effectiveness for the treatment of SAM, healthy children with a WHZ-score between −3 and −1 and aged 3 – 5 years of age were recruited from 2 kindergartens in HaNam province, 40 km to the south of Hanoi. Children with known food allergies or hepatic problems were excluded from the study.

This study was conducted according to the guidelines laid down in the Declaration of Helsinki and all procedures involving human subjects/patients were approved by the [name of the ethics committee removed for blinding]. Written informed consent was obtained from all subjects.

Children were served two meals with RUTF for 5 (school) days per week. The RUTF was given at 9:30 am and at 3 pm, replacing the normal snack meal which is provided at those times, and children were given 60 minutes each time to eat the RUTF under supervision and assistance if necessary. Children were offered 1 sachet of RUTF/meal, providing approximately 1000 kcal / day (1 sachet = 500–530 kcal). Children were gathered in two different class rooms (one allocated for each RUTF), where they were provided with either Plumpy’nut^®^ or HEBI, depending on randomization. During each meal, intake was recorded and information collected on amount eaten and wasted and duration of eating. Safe drinking water was freely available. Reluctance to eat the product was defined as refusing to eat the product the first time the teacher presented it, but after encouragement by the teacher, the child tried the offered food. Refusal to eat the product meant that the child completely refused the offered product even after being encouraged twice by the teacher.

#### Acceptability and organoleptic qualities

Organoleptic qualities (color, smell, taste, palatability and hardness) of the 2 RUTFs were assessed using a simple pictogram questionnaire appropriate for the age group, with smiley’s representing bad, neutral or good. The questionnaire was filled the last school day of each week, i.e. every Friday, when the children had completed a full week of eating the RUTF, with trained field assistants using simple language to avoid mistakes in interpretation. Acceptability of the product was defined as consumption of more than 75% of the offered food within 1 hour (meal acceptance) and consumption of more than 75% of the offered food for more than 75% of the days on trial (overall acceptance). In addition to the amount of RUTF eaten eating patterns were assessed. Teachers recorded if children refused completely the RUTF or showed reluctance to try the product at first and only ate the offered RUTF after encouragement.

#### Anthropometry and morbidity data

At baseline, data on weight, height and mid upper arm circumference (MUAC) were collected. Child’s weight was measured to the nearest 0.1 kg on a portable digital scale (SECA, Germany) with children wearing light clothes following standard procedures. The standing height of the children was measured without shoes to the nearest 0.1 cm using a wooden height board (UNICEF, USA). MUAC was measured to the nearest 0.1 cm at the midpoint of the left arm between the acromion process and the tip of the olecranon with a standard MUAC tape (UNICEF). Weight measurements were repeated weekly, MUAC was measured at end of week 2 and at end-point, and height was measured again at end-point. Anthropometry was done by the same, trained field assistants at all time points, using standard methods, and wearing light summer clothes without shoes. Weight was measured with a SECA scale (0.1 kg precision), MUAC using standard UNICEF tape measures, and height using a Stanley measuring tape (0.1 cm precision). Illness symptoms and signs, such as nausea, vomiting, diarrhea and the presence of any skin rash were assessed by the teachers, by questioning the mothers about their children’ health status and by observing the children during the hour following each meal. All symptoms were recorded daily on a standard data collection sheet.

#### Sample size determination

The main outcome of the study was the acceptance of one or both products by the children. As it was difficult to find general guidelines for when a RUTF is considered acceptable, overall acceptance of a product was defined a priori as children eating >50% of the offered product, with >75% of the product being consumed being regarded as good acceptability. Furthermore, we defined a clinical relevant difference between the 2 products as a difference in consumption of >20%. Hence, a sample size of at least 50 children was needed to detect a relevant difference between 50% and 70% of consumption of each product. This sample size would also allow the detection of 0.1 z-score difference over the intervention period, assuming a SD of 0.80, with a power of 0.80 and a significance of 0.05. As we expected *a priori* a high reluctance to eat PlumpyNut, based on the earlier report from Cambodia, we recruited all 67 children eligible in the 2 schools.

#### Statistical analyses

Summary enrollment characteristics were calculated as means ± SD for continuous measures. Anthropometric indices were calculated using WHO 2006 standards (ANTHRO version 3.2.2 January 2011) and expressed as z-scores for weight-for-height (WHZ), weight-for-age (WAZ) and height-for-age (HAZ). Weight gain was determined by calculating the change in weight during each week of the study, and overall weight gain was expressed as g/kg body weight per day, calculated over the duration of the study. Because of the introduction of a new product, the prevalences of nausea, vomiting, rash, and diarrhea were measured during the intervention to assess side effects, but the prevalences were too low to allow for meaningful statistical analysis. Comparison of continuous parameters was made using ANCOVA, controlling for baseline values, and paired t-test. Ordinal data were analyzed using a Wilcoxon signed rank test (SPSS 15.0 for Windows, Chicago, IL). Statistical significance was defined as P < 0.05.

## Results

Of the 67 children recruited for the study, one child dropped out (Group 2, Figure 2) as the parents did not bring the child to school frequently. At baseline, mean WAZ, HAZ and WHZ were −2.12 (± 0.63), -1.55 (± 0.87) and −1.81 (± 0.49) respectively, characterizing the overall study population sample as underweight but not acutely malnourished. However, 22.7% (n = 15) were classified as Moderate Acute Malnutrition (MAM) i.e. with a WHZ between −3 and −2 and 3% (n = 2) of the children had SAM (one in each group).

More Plumpy’nut^®^ than HEBI was consumed during the 4 weeks of intervention (Table 2), both in total amount eaten as well as percentage of RUTF offered, with Plumpy’nut^®^ intake being 78.7 (± 11.4) g/meal (85.6% of the overall quantity offered (92 g)) compared to 70.8 (± 14.0) g/meal HEBI (70.8% of the overall quantity offered (100 g)) (P < 0.001). Overall, children ate more RUTF in the second week than in the first week of any product as shown in Table 2 (P < 0.05 and P < 0.01 for Plumpy’nut^®^ and HEBI respectively).

**Table 2:** **Consumption and eating patterns of the RUTF consumption** (**for both groups**)

			**Week 1 of the RUTF consumption**	**Week 2 of the RUTF consumption**
**Consumption**^**1**^	Amount RUTF eaten (g ± SD, % offered in brackets)	Plumpy’nut^®^	77.1 ± 14.4	80.6 ± 10.7
			(83.8%) ^a^	(87.6%) ^b^
		HEBI	68.0 ± 14.2	73.2 ± 15.2
			(68.0%) ^c^	(73.2%) ^d^
**Eating patterns**	Reluctance to eat offered RUTF (N, (mean, (% of the children))	Plumpy’nut^®^	13 (19.7%)^a^	3 (4.5%)^b^
		HEBI	8 (12.1%)^c^	2 (3%)^b^
	Mean duration of the meal (Min, mean, (SD))	Plumpy’nut^®^	40.6 (± 11.7)^a^	36.9 (± 11.5)^b^
		HEBI	42.9 (± 11.5)^c^	41.2 (± 10.7)^d^

^1^ Cells with different letters differ significantly from each other (P < 0.05).

Interestingly, although overall intake in the Plumpy’nut^®^ group was higher, children also showed a higher reluctance towards Plumpy’nut^®^ than to HEBI (P < 0.05), with overall reluctance to both products being high especially during the first week (Table 2). For both RUTF, children spent less time to eat them during the morning meal than the afternoon meal, with a mean duration of 39.1 (± 9.8) min and 41.5 (± 10.7) min respectively (P < 0.05). Also, they spent less time the 2nd week of consumption compared to the first week (P < 0.05, Table 2). Children of both group spent less time eating Plumpy’nut^®^ compared to HEBI ( 38.6 (± 11.1) min and 42.0 (± 10.8) min respectively, P < 0.05).

Overall, both RUTFs scored high for organoleptic qualities (i.e. for color, smell, taste, palatability), with more than 75% children scoring ‘good’ for both products. However Plumpy’nut^®^ scored significantly higher for palatability (P < 0.05, Table 3), and HEBI scored higher for hardness (P < 0.01). The latter was according to expectations, as the local RUTF is a compressed product (bar) compared to the spread-like Plumpy’nut^®^.

**Table 3:** **Sensory scores given by the children for both types of RUTF**^**1**^

		**Smell**	**Color**	**Taste**	**Palatability**^**2**^	**Hardness**^**2**^
Plumpy’nut^®^	**Week 1**	2.89 (0.41)	2.86 (0.40)	2.87 (0.38)	2.84 (0.41)	1.21 (0.57)
	(N = 63)					
	**Week 2**	2.80 (0.48)	2.84 (0.44)	2.86 (0.43)	2.88 (0.42)	1.08 (0.37)
	(N = 64)					
**HEBI**	**Week 1**	2.75 (0.59)	2.81 (0.53)	2.81 (0.53)	2.70 (0.61)	1.58 (0.90)
	(N = 64)					
	**Week 2**	2.79 (0.57)	2.53 (0.77)	2.76 (0.58)	2.67 (0.64)	1.59 (0.82)
	(N = 66)					

^1^ Sensory qualities were scored on a range from 1 – 3 (bad, normal, good). Scores are given as mean (SD).

^2^ Plumpy’nut^®^ scored significantly higher for overall palatability (week 1 + 2 combined, P < 0.05) as compared to HEBI, whereas HEBI scored significantly higher for overall hardness (but for not acceptability) of the product (week 1 + 2 combined, P < 0.01).

There were significant increases in all anthropometrical indices over the 4 week intervention period (Table 4) with no difference between the 2 RUTF (P > 0.05). Weight increased on average 0.64 (± 0.27) Kg, with similar weight increases over the 2 week interventions for HEBI and Plumpy’nut^®^ (0.32 kg/2 weeks for both, P > 0.2). The mean rate of weight gain was 1.87 (± 0.81) g/kg body weight per day. Height increased on average 0.7 (± 0.5) cm over the 4 week study period, more than expected, which is also reflected in significant higher WAZ, HAZ and WHZ scores at the end of the study, with increases of 0.34, 0.05 and 0.48 Z-scores respectively (P < 0.01 for all). MUAC increased by 0.5 (± 0.3) cm (P < 0.001) over the intervention period.

**Table 4:** Anthropometric characteristics of the children who completed the study at baseline and after receiving RUTF 5 times per week for 4 weeks

	**Baseline**	**End point**	**P**-**value**
	**Mean (SD)**	**Mean (SD)**	
Weight (kg)	12.46 (± 1.56)	13.1 (± 1.59)	<0.001
Height (cm)	96.49 (± 6.61)	97.14 (± 6.65)	<0.001
WAZ (z-score)	−2.12 (± 0.63)	−1.77 (± 0.61)	<0.001
HAZ (z-score)	−1.55 (±0.87)	−1.50 (± 0.87)	0.003
WHZ (z-score)	−1.81 (±0.49)	−1.33 (± 0.46)	<0.001
MUAC (cm)	13.80 (± 0.56)	14.32 (± 0.64)	<0.001

## Discussion

This study shows that a locally produced RUTF (HEBI) was highly acceptable for children to eat. The formulation of HEBI has been developed on basis of mung bean cake (Banh Dau Xanh), which is popular among the Vietnamese population, thereby increasing the likelihood for acceptance, and facilitating local production. This study also shows that in contrast to an earlier report from Cambodia, Plumpy’nut^®^ appears to be accepted by children in Vietnam. Indeed, Plumpy’nut^®^ scored higher than HEBI for the organoleptic qualities as scored by the children. Surprisingly, the acceptability problem appeared to reside more with the adults as it was noticed the teachers were reluctant to give the Plumpy’nut^®^ paste to the children at first, because it was so different from the Vietnamese tastes and habits, whereas the local RUTF was immediately approved and understood by the teachers. But after information and communication were successfully given before the start of the project, parents, school teachers, local authorities, and health staff became highly interested in both products, which is in contrast to the Cambodian study. Thus this study confirms the previous finding of the Cambodian study, which highlighted that “accepting or refusing […] is less a personal choice coming from the child alone but more a collective outcome. […] The notion of acceptability is the result of a social commitment which, for working more satisfactorily, must encourage the active participation of various social actors” [10].

Based on the definition of acceptability by AFNOR standards [12], both RUTF were highly accepted according to their organoleptic properties. Based on the protocol of the study (with good acceptability defined as a consumption of more than 75% of the offered meal), Plumpy’nut^®^ was accepted by the children with more than 85% of the overall offered quantity consumed. However, the local RUTF with 71% of the overall quantity eaten fell below this pre-set target. Nevertheless, the amount of local RUTF consumed was still high and can be considered promising. One likely reason for the higher consumption of Plumpy’nut^®^ compared to the local RUTF was the dryness of the local RUTF bar compared to the spread-like Plumpy’nut^®^. The study was indeed conducted in July, the warmest month of year with average temperatures of 35–39°C. Even though water was freely available for the children, several children complained of being thirsty, a feature reported earlier after RUTF consumption [13]. Furthermore, the package of the Plumpy’nut^®^ was more attractive than that of the local product, with color and drawings on the package. The children loved to play with this package during eating, and this may even have biased the reporting of the color of the product, with children referring to the packaging and not the product itself. Since the trial, slight changes have been made to the composition of the local RUTF to make it less dry, and the packaging has been improved, addressing the two most obvious aspects that determined the children’s relative experience of the products.

Interestingly, there was a significant increase in the amount of RUTF consumed from week 1 to week 2. Children showed more reluctance to eat Plumpy’nut^®^ in the first week, as the paste-like form, consumed directly from a bag is not customary in Vietnam. This reluctance decreased in the second week. This probably signifies a familiarization with the product and highlights the importance of providing guidance and product demonstration during the initial phase of using RUTF in the treatment of SAM.

The current study was performed in a school setting with teachers present. This resulted in adults helping the children to eat, and this may have increased the overall amount of RUTF consumed. However, in view of the use of RUTF in the IMAM program, this would actually closely represent a situation of a motivated well-instructed mother feeding her malnourished child. The harder consistency of the HEBI bar in comparison to the spread-like Plumpy’nut^®^ may make it less suitable for children under 12 months of age. Therefore, a spread-like alternative is currently being developed.

Another important finding of the study is that both products resulted in significant weight and height changes over the 4 week intervention. In this underweight but not acutely malnourished population, the intervention resulted in a sharp increase in WAZ and WHZ scores, even though the total duration of the intervention was only 4 weeks. This can be considered an indication of the potential effectiveness of RUTF in malnourished Vietnamese children. If the results of the present study are confirmed in an equivalence trial, the Vietnamese RUTF could be included in the National Plan for the Integrated Management of Acute Malnutrition which is currently being developed. Interestingly, there was also an increase in HAZ-score, even though the increase was modest (0.05 z-score). But given the short duration of the intervention, we had not expected to find significant effects on length growth in this study, and this finding merits extra attention. This increase in HAZ-score clearly shows that increases in height are possible even in children with an average age of 4 yrs [14], and indicates that supplementary feeding with macro- and micronutrients in normal rural Vietnamese schoolchildren can significantly improve growth and nutritional status. However, for this increase in macro- and micronutrient intake, a specialized, highly concentrated food product such as RUTF is perhaps not necessary, and a lower cost, more sustainable food intervention might result in improvements in weight and height also, although data on this is currently lacking and requires further investigation. Furthermore, this study also indicates that more targeted programs providing supplementary foods for children with moderate malnutrition, even though they are older than 2 years, are very likely to reduce the rate of stunting.

In order to finalize the development of the local RUTF, and ascertain the product can be used, licensed and certified as a complete and effective nutritional intervention tool in programs such as IMAM, an effectiveness trial is currently being conducted in children with SAM and MAM.

## Conclusions

This study showed that a local produced RUTF was acceptable by pre-school children. Consumption of the local produced RUTF and a commercial available RUTF resulted in significant gains in anthropometry. Further improvements of the local product are needed, and the impact on weight gain and recovery from SAM needs to be tested before the product can be used for the integrated management of acute malnutrition.

## Competing interests

The authors declare that they have no competing interests.

## Authors’ contribution

TTN, MN, RM, JB and FTW conceived the study. TTN, MN and FTW were responsible for supervision of the field work. MN, DTBH, NHM, FTW were responsible for the development of the RUTF. The first draft of the manuscript was written by TTN, MN, JB and FTW. All authors read and commented on the draft manuscript and approved the final manuscript. All authors read and approved the final manuscript.

## References

[B1] WHO, WFP, SCN and UNICEF Community-based management of severe acute malnutrition: A Joint Statement by the World Health Organization, the World Food Programme, the United Nations Standing Committee on Nutrition and the United Nations Children’s Fund 2007 Geneva http://www.unicef.org/publications/files/Community_Based_Management_of_Sever_Acute__Malnutirtion.pdf

[B2] Linneman Z Matilsky D Ndekha M Manary MJ Maleta K Manary MJ A large-scale operational study of home-based therapy with ready-to-use therapeutic food in childhood malnutrition in Malawi Matern Child Nutr 2007 3 206 215 10.1111/j.1740-8709.2007.00095.x 17539889 PMC6860523

[B3] Manary MJ Local production and provision of ready-to-use therapeutic food (RUTF) spread for the treatment of severe childhood malnutrition Food Nutr Bull 2006 27 S83 89 17076214 10.1177/15648265060273S305

[B4] Briend A Lacsala R Prudhon C Mounier B Grellety Y Golden MH Ready-to-use therapeutic food for treatment of marasmus Lancet 1999 353 1767 1768 10.1016/S0140-6736(99)01078-8 10347999

[B5] Diop El DN Ndour MM Briend A Wade S Comparison of the Efficacy of a Solid Ready to Use Food and a Liquid Milk-based Diet for the Rehabilitation of Severely Malnourished Children: A Randomized Trial Am J Clin Nutr 2003 78 302 307 12885713 10.1093/ajcn/78.2.302

[B6] Ciliberto MA Sandige H Ndekha MJ Ashorn P Briend A Ciliberto HM Manary MJ Comparison of home-based therapy with ready-to-use therapeutic food with standard therapy in the treatment of malnourished Malawian children: a controlled, clinical effectiveness trial Am J Clin Nutr 2005 81 864 870 15817865 10.1093/ajcn/81.4.864

[B7] Navarro-Colorado CLS Clinical trial of BP100 vs F100 milk for rehabilitation of severe malnutrition Field Exchange 2005 24 22 24

[B8] WHO Manual for the health care of children in humanitarian emergencies 2008 Geneva http://whqlibdoc.who.int/publications/2008/9789241596879_eng.pdf 23785739

[B9] UNICEF Tracking progress on child and maternal nutrition: A survival and development priority 2009 http://www.unicef.org/publications/files/Tracking_Progress_on_Child_and_Maternal_Nutrition_EN_110309.pdf

[B10] Bourdier F Socio-anthropological investigation related to the acceptability of Plumpy’nut in Cambodia, Research document 2009 Paris, France: Institut de Recherche pour le Développement

[B11] National Institute of Nutrition Prevelence of children under 5 years old undernutrition by severity – 2010 - by 6 ecological region 2010 http://viendinhduong.vn/FileUpload/Documents/Ty%20le_SDD%20TE_%20nam%202010.pdf

[B12] Sztrygler F SSHA and ISHA Evaluation sensorielle, manuel méthodologique 1990 Paris: Lavoisier Tec&Doc 309

[B13] WHO National guidelines for the management of acute malnutrition among children under five and pregnant and lactating women 2010

[B14] Golden MH Is complete catch-up possible for stunted malnourished children? Eur J Clin Nutr 1994 48 Suppl 1 S58 70 8005092

